# Influence of Trabecular Geometry on Scaffold Mechanical Behavior and MG-63 Cell Viability

**DOI:** 10.3390/ma16062342

**Published:** 2023-03-15

**Authors:** Maria Laura Gatto, Giorgia Cerqueni, Michele Furlani, Nicole Riberti, Emanuele Tognoli, Lucia Denti, Francesco Leonardi, Alessandra Giuliani, Monica Mattioli-Belmonte, Paolo Mengucci

**Affiliations:** 1 Department DIISM, Università Politecnica Delle Marche, Via Brecce Bianche 12, 60131 Ancona, Italy; m.l.gatto@univpm.it; 2 Department DISCLIMO & UdR INSTM, Università Politecnica Delle Marche, Via Tronto 10/a, 60126 Ancona, Italy; g.cerqueni@univpm.it (G.C.); m.mattioli@univpm.it (M.M.-B.); 3 Department DISCO, Università Politecnica Delle Marche, Via Brecce Bianche 12, 60131 Ancona, Italy; m.furlani@pm.univpm.it (M.F.); a.giuliani@univpm.it (A.G.); 4 Neurosciences Imaging and Clinical Sciences Department, University of Chieti-Pescara, 66100 Chieti, Italy; nicole.riberti@unich.it; 5 Department of Engineering “Enzo Ferrari”, Università di Modena e Reggio Emilia, Via Vivarelli 10, 41125 Modena, Italy; emanuele.tognoli@unimore.it (E.T.); lucia.denti@unimore.it (L.D.); 6 PuntoZero 3D S.r.l., Via Larga 19, 20122 Milano, Italy; francesco.leonardi@puntozero3d.com; 7 Department SIMAU & UdR INSTM, Università Politecnica Delle Marche, Via Brecce Bianche 12, 60131 Ancona, Italy

**Keywords:** trabecular geometry, generative design, tissue engineering, vat photopolymerization, biomechanical performances, morphometric parameters

## Abstract

In a scaffold-based approach for bone tissue regeneration, the control over morphometry allows for balancing scaffold biomechanical performances. In this experimental work, trabecular geometry was obtained by a generative design process, and scaffolds were manufactured by vat photopolymerization with 60% (P60), 70% (P70) and 80% (P80) total porosity. The mechanical and biological performances of the produced scaffolds were investigated, and the results were correlated with morphometric parameters, aiming to investigate the influence of trabecular geometry on the elastic modulus, the ultimate compressive strength of scaffolds and MG-63 human osteosarcoma cell viability. The results showed that P60 trabecular geometry allows for matching the mechanical requirements of human mandibular trabecular bone. From the statistical analysis, a general trend can be inferred, suggesting strut thickness, the degree of anisotropy, connectivity density and specific surface as the main morphometric parameters influencing the biomechanical behavior of trabecular scaffolds, in the perspective of tissue engineering applications.

## 1. Introduction

In the field of bone tissue engineering, a scaffold-based approach allows for preserving tissue functionalities by means of synthetic substitutes [1]. Controlling scaffold morphometric parameters allows to balance biomechanical performances [2,3], affecting the time and quality of regeneration [4]. Appropriate design of the porous network and total porosity should promote cell ingrowth and proliferation [5] and avoid stress-shielding phenomena in the host tissue [6].

A modern approach to scaffold design is based on the use of hierarchical structures, created by the repetition of a unit cell of well-defined geometry and properties, and on the possibility to predict the properties of the scaffold from the known properties of the unit cell [7,8]. However, the unit cell approach produces architectures that do not truly mimic natural bone histomorphometry. To overcome this limitation, biomimetic scaffolds with a trabecular structure, mimicking natural bone, are created by a generative design approach. The generative design allows for obtaining complex shapes by an automatic modeling process [9,10], and results can be easily customized by interactively modifying morphometric parameters [11,12,13,14].

Nevertheless, in addition to the design, the type of biomaterial and manufacturing process plays a pivotal role to obtain suitable trabecular scaffolds. The accurate control of material distribution can be efficiently obtained by a layer-by-layer production strategy typical of the additive manufacturing (AM) process. Among various AM technologies, Vat photopolymerization (VPP) acquired broad attention in dentistry and biomedical engineering, encompassing nervous, cardiovascular, musculoskeletal and respiratory system applications [15].VPP uses photopolymerization to create solid objects from a vat of liquid resins under light irradiation [16] and involves the following post-processing steps: (1) the removal of excess resin from the manufactured part; (2) post-curing in a UV oven; (3) the removal of supports and (4) cleaning. The advantages presented by VPP are connected to high resolution (approximately 150 μm), printing speed, smooth final finishing, moderate cost and low waste materials (uncured resin is reusable) [15].

A wide range of resins have been developed for VPP, and many of them are commercially available. Such resins are normally composed of multi-functional monomers based on methacrylate or acrylic esters. However, the actual chemical composition is often confidential and not provided by manufacturers; thus, the physicochemical and mechanical properties as well as the cytotoxicity remain unclear [16].

DS3000 (DWS Srl) is a commercial biocompatible resin developed for medical applications requiring a limited contact time with the body. In dentistry applications, DS3000 was used to create a surgical splint for the planning of jaw repositioning surgery [17]. Additionally, customized dental impressions were manufactured in DS3000 to facilitate the visualization of margins preparation for fillings [18]. However, DS3000 manufactured by VPP was also used for 3D architectures with multidisciplinary potential applications, ranging from biomimetic scaffolds for cell culture to microfluidics [19,20]. Han et al. [19] created an in vitro model of a bone metastasis microenvironment in DS3000, reproducing the trabecular architecture defined from X-ray micro-computed tomography scan images. Scaffold cytocompatibility was proved to be suitable for cultivating and differentiating mesenchymal stem cells and breast metastatic tumor cells. Although this bone model does not fully replicate the real bone niche complexity, Han et al. found that it possessed the potential to address a wide range of biological issues related to complex cell–bone interactions [19].

Starting from the findings of Han et al. [19], our work relates the morphometric parameters of trabecular structures with their mechanical and biological behavior, with the aim of finding the geometric features which mainly affect the scaffold biomechanical response in the perspective of bone tissue regeneration. As in Han et al. [19], the low-cost biocompatible commercial material DS3000 was used in this study for the manufacturing of our trabecular structures.

In this experimental study, DS3000 trabecular scaffolds were produced by the vat photopolymerization technique. Trabecular geometry was obtained by a generative design process, modulating the number of seeds and strut thickness to achieve the target scaffold porosity and pore size. Three different trabecular scaffolds were manufactured by VPP in DS3000, with 60%, 70% and 80% of total porosity and increasing mean pore size. The performances of the three trabecular architectures were evaluated through the systematic quantification of morphometry as well as mechanical and biological properties. Statistical analysis evidenced a general trend of the morphometric parameters mainly influencing the scaffold biomechanical behavior.

## 2. Materials and Methods

### 2.1. Scaffold Generative Design

**Figure 1:**
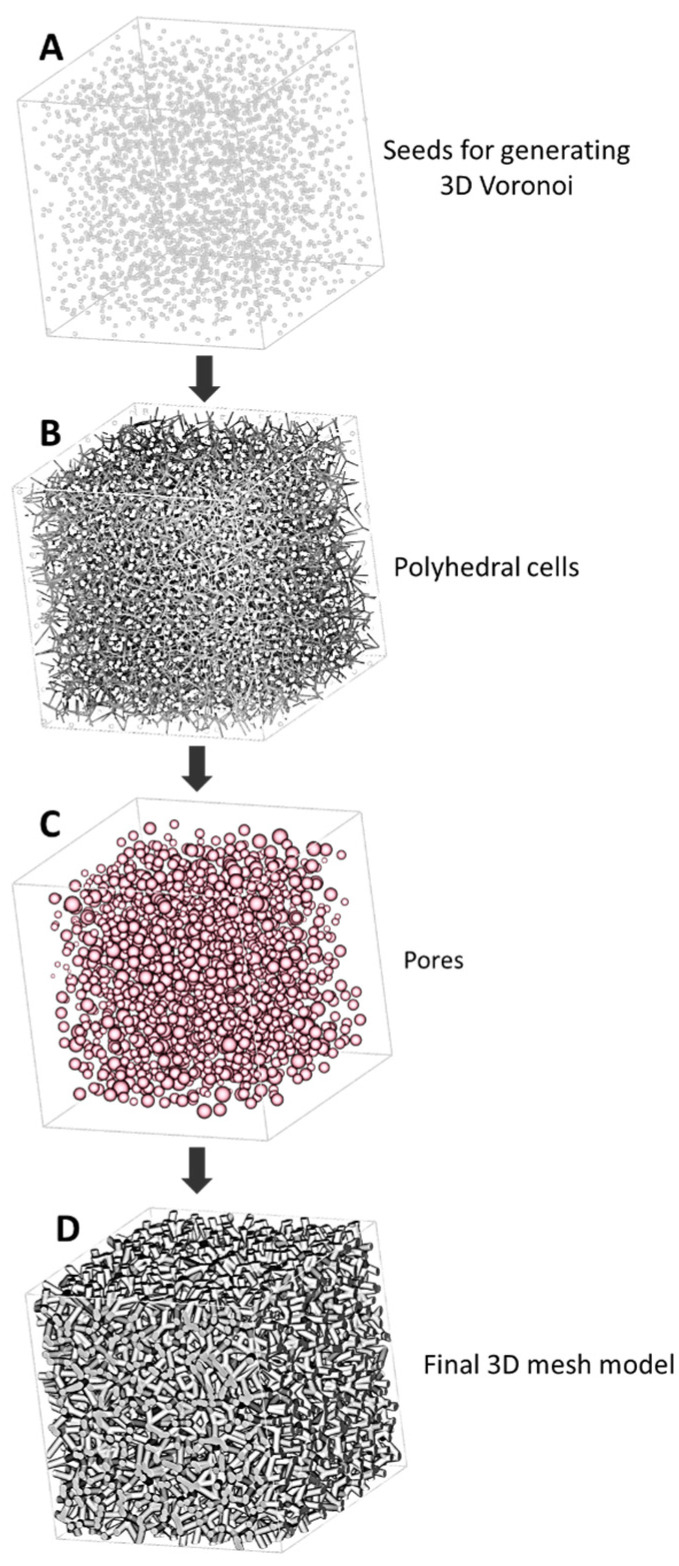
Generative design flowchart for designing trabecular scaffold, based on Voronoi diagram: (**A**) discrete set of points (seeds) randomly distributed in the bounding box volume; (**B**) 3D Voronoi set of polyhedral cells; (**C**) pores generation-pores were represented as spheres with equivalent volume; and (**D**) final 3D mesh model.

Trabecular scaffolds were created by a generative design process (Figure 1), implemented in nTopology (nTopology Inc., New York, NY, USA). A generative design flowchart (Figure 1) allowed to design Voronoi-based trabecular scaffolds by controlling the porosity and the mean pore size. Synthetically, the main steps, depicted in Figure 1, were: (a) a discrete set of points (seeds) distributed in a bounding box volume was randomly generated (Figure 1A), (b) from such a set of seeds, the 3D Voronoi set of polyhedral cells (American Type Culture Collection, CRL-1427™) was developed (Figure 1B), (c) scaffold pores were generated (Figure 1C) according to the number of seeds and centered at the centroid of the polyhedral cell. Pores were delimited by the network of solid beams (struts) built along the edges of the polyhedral cells. The two input parameters, number of seeds and strut thickness, can be interactively modified in order to control the output values of porosity and mean pore size, as extensively described by Fantini et al. [7,21]. These output parameters have an important impact on bone regeneration [21]. The final 3D mesh model obtained by this procedure is the trabecular structure shown in Figure 1D. The closed 3D mesh model of the trabecular scaffold, with porous and interconnected architecture, was finally saved in STL format. To test the pore interconnectivity, a negative model of the scaffold volume was created and meshed to verify any single component and/or isolated pore. 

A cubic trabecular scaffold 20 mm side (bounding box volume 8000 mm^3^) with three different porosity values (60, 70 and 80%) was designed following the steps illustrated in Figure 1. To complete the scaffold generation process, the smoothing of the final 3D mesh model of the P60 scaffold was carried out by the Gaussian Image Kernel algorithm. The values of target porosity, target mean pores size, number of seeds, strut thickness and average strut length of scaffolds are reported in Table 1. From here on, scaffolds are named with reference to their values of porosity (P). As an example, the scaffold with 60% porosity is named as P60.

### 2.2. Scaffold Manufacturing

DS3000 trabecular scaffolds were produced by the Vat photopolymerization (VPP) technique using a DWS 029X machine (DWS System, Thiene, Italy) operating with a 405 nm-wavelength laser. The optimized printing parameters used for the manufacturing are reported in Table 2.

### 2.3. Structural Characterization

Scanning electron microscopy (SEM) observations by a Tescan Vega 3 (Brno, Czech Republic) were carried out to investigate the surface morphology of all trabecular scaffolds (P60, P70 and P80). SEM observation was performed on a scaffold seeded with cells (P60), after 24 h from MG-63 cell seeding on the top surface and after 7 days of culturing in the scaffold core, after cutting a sample at about 5 mm height. Furthermore, strut failures of P60 scaffold geometry were observed with SEM after mechanical compression tests. This work is focused on the study of the biological and mechanical performances of the P60 trabecular geometry because the mechanical tests performed on all samples, reported in Section 3.2, provided the ultimate compressive strength value of the P60 geometry within the range of values of the human mandible [22]. Conversely, the ultimate compressive strength values of P70 and P80 architectures fall outside this range (Table 3). Therefore, only the P60 geometry was fully characterized, as reported in the following.

X-ray computed microtomography (XµCT) analysis was performed on P60, P70 and P80 trabecular scaffolds before and after mechanical testing. A Bruker Skyscan 1174 tomographic system (Kontich, Belgium) was used to obtain scaffold projections at V = 50 kV and I = 800 µA, with the following experimental settings: pixel size = 22.23 μm; rotation step = 0.4° for 360°; exposure time per projection = 1.6 s. Projections were processed in a stack of cross-sectional slices by the SkyScan reconstruction program NRecon, with the following conditions: smoothing = 3; ring artefact reduction = 6; beam hardening correction = 30%. Additionally, possible misalignment during acquisition was compensated. A 3D reconstruction of P60, P70 and P80 representative volume (10 × 10 × 10 mm^3^) was obtained for material distribution (strut map) and porous network (pore map), using, respectively the Thickness map and Compute ambient occlusion functions of Avizo software (vers. 2019.1, Thermo Fisher Scientific, Waltham, MA, USA). Furthermore, a 3D reconstruction of the P60 scaffold geometry after a mechanical compression test (Table 3) was performed with the deep learning module of Dragonfly software (Version 2022.1; Object Research Systems, Montreal, QC, Canada), allowing the volumetric rendering of strut failures.

Morphometric parameters such as specific surface (mm^−1^), average strut thickness (mm), average pore size (mm) and total porosity (%) of trabecular scaffolds were quantified pre- and post-mechanical tests, employing SkyScan CT-analyzer software (Bruker). In addition to these parameters, the connectivity density and the degree of anisotropy were analyzed to correlate morphometry with the biomechanical behavior of scaffolds (see Section 2.6). Connectivity density (mm^−3^) indicates the number of connections between trabecular structures per unit volume, representing a global measure of structure interconnectivity, which gives higher values for better-connected structures and lower values for poorly connected structures. The degree of anisotropy is a measure of 3D symmetry, indicating the presence or absence of the preferential alignment of structures along a particular directional axis. Degree of anisotropy values varies between 0, corresponding to the perfect isotropy of the scaffold structure, and 1, representing structures perfectly oriented in agreement with a single plane or axis.

Phase-contrast XµCT (PhC-XµCT) measurements were carried out at the SYRMEP beamline of the ELETTRA Synchrotron Radiation facility (Trieste, Italy), using a white X-ray beam with 17 keV peak energy, a sample-to-detector distance of 100 mm and a pixel size of 0.9 μm. Analysis was performed on P60 trabecular scaffold in the following conditions: as-built, after 7 days of incubation with only culture medium and after 7 days of incubation with MG-63 human osteosarcoma cells, in order to assess the effect of cells on surface topology. PhC-XµCT imaging is based on the phase variation of X-ray electromagnetic waves after interaction with samples. For X-rays, the refractive index (n) of the material is a complex number (Equation (1)):

n = 1 − δ + iβ(1)

where δ is the decrement of the real part of the complex refractive index n, whereas the imaginary part β is the extinction coefficient, which describes the material absorption. For the polymer scaffold, the soft tissues and cells used in the biological experiments of this work, δ, is about three orders of magnitude higher than β, thus resulting in higher sensitivity of the phase-contrast approach with respect to the absorption contrast of conventional XµCT [23]. Segment length maps of the scaffold surface pattern were extracted from 3D surface models of P60 with Dragonfly software (Vers. 2022.1; Object Research Systems, Montreal, QC, Canada). The quantification of the average segment length of the P60 surface pattern in the following conditions: as built, 7 days culture medium and 7 days cells, was obtained from six segment length maps for each condition. Each map was derived from a volume of interest of 500 µm^3^.

### 2.4. Mechanical Compression Tests

The mechanical performances of P60, P70 and P80 trabecular scaffolds were investigated by uniaxial compressive tests carried out by an Instron 5567 system (Norwood, MA, USA) with 1 kN load cell, 0.5 mm/min speed and preload of 2 N. For each scaffold geometry, five cubic samples 20 × 20 × 20 mm^3^ were tested. The results of the compressive tests were plotted as stress–strain curves. From the stress–strain curves, the Young modulus (E_S_) and nominal ultimate compressive strength of the trabecular scaffolds at 40% of compressive strain (σ_UCS_) were calculated.

### 2.5. Biological Tests

#### 2.5.1. Cell Culture

MG-63 human osteosarcoma cells (ATCC, CRL-1427) were maintained in Dulbecco Modified Eagle’s Medium (H-DMEM, Corning Inc., Somerville, MA, USA, D6429), 1% penicillin–streptomycin (Thermo Fisher Scientific, 15140122) and 10% FBS (Corning Inc., 35-079-CV) in a humidified incubator at 37 °C and 5% CO_2_, refreshing the medium every 3 days. For passaging, trypsin/EDTA (trypsin 0.05%– EDTA 0.02% in PBS, Sigma-Aldrich, USAT4174) was used.

#### 2.5.2. Sterilization and Conditioning

P60 scaffolds (10 × 10 × 10 mm^3^) were sterilized with EtOH 70% for 30 min, washed three times with PBS and UV irradiated for 30 min on each side. After sterilization, the samples were conditioned with H-DMEM with 10% FBS and 1% penicillin/streptomycin overnight.

#### 2.5.3. MG-63 Seeding

P60 scaffolds were placed in wells of 12 wells/plate, and, after preconditioning with culture medium, 8 × 10^4^ MG63/scaffold were seeded. Samples were cultured at 37 °C and 5% CO_2_ for 24 h, 72 h and 7 d. The medium was replaced every two days. MG63 viability was assessed by MTT assay (Section 2.5.4), whereas the MG63 cell morphology was observed by SEM (Section 2.5.5). Three experimental sets were set up and each assay was performed in triplicate.

#### 2.5.4. MTT Assay

The metabolically active MG63 was assayed by MTT (3-dimethylthiazol-2,5-diiphenyltetrazolium bromide, Sigma–Aldrich, M5655) according to the manufacturer’s instructions. Briefly, the MTT stock solution (5 mg/mL) was diluted 1:10 in cell culture medium, composed of H-DMEM without phenol red, and incubated at 37 °C for 3 h. After incubation, the medium was removed and DMSO was added to each well to dissolve the purple formazan crystals. Then, the absorbance was quantified by spectrophotometry (MultiskanGo, Thermo Scientific, Wilmington, DE, USA), monitoring the absorbance at 570 nm with reference wavelength at 650 nm.

#### 2.5.5. Cell Morphology

Samples with cells were fixed in 2% glutaraldehyde (MERCK, 4239) in 0.1 M sodium cacodylate buffer (Sigma, C-0250), followed by washes in 7% sucrose in 0.1 M cacodylate buffer and post-fixation in 1% osmium tetroxide (Electron Microscopy Sciences, 12310) in 0.1 M sodium cacodylate buffer. Complete dehydration was achieved in graded alcohol series (25%, 50%, 70%, 80%, 95% and 100%) and Critical Point Dry was performed with hexamethyldisilane (HMDS, Sigma Aldrich, St. Louis, MO, USA, 440191). Afterwards, the samples were gold-sputtered by the Edwards Sputter Coater B150S equipment and observed by SEM Tescan Vega 3. 

#### 2.5.6. Statistical Analysis

The statistical significance of the results was evaluated by GraphPad Prism Software (v. 9.1.1), using two-way ANOVA with repeated measurements. Then, Tukey’s post hoc test was carried out to highlight the main factors determining data variability. Statistical significance was set at **** *p* < 0.0001; *** *p* < 0.001; ** *p* < 0.01.

### 2.6. Correlation between Morphometry and Biomechanical Properties

Statistical analysis was conducted to find the correlation of morphometric parameters of trabecular scaffolds with mechanical values and MG63 viability. The Spearman’s correlation coefficient (SCC), also referred to as rs, was selected as the correlation coefficient since any assumption on the distribution of parameters collected as study data cannot be made (only five repetitions for each parameter). Rs was evaluated to measure the linear correlation between each morphometric feature, obtained by XµCT, and the mechanical and biological features resulting from the scaffold tests. A correlation matrix was obtained from SCC evaluation using GraphPad Prism Software (v.9.1.1).

## 3. Results

### 3.1. SEM Analysis

**Figure 2:**
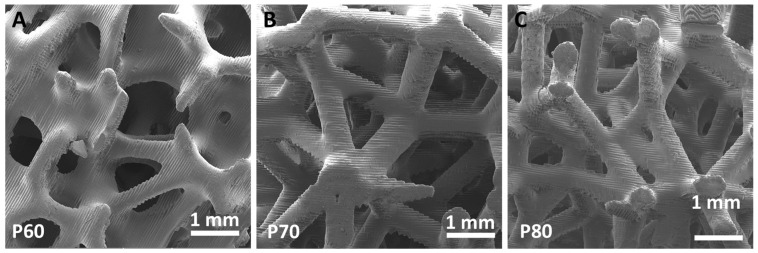
SEM micrographs of P60 (**A**), P70 (**B**) and P80 (**C**) trabecular scaffolds.

SEM observations of scaffolds obtained by generative design processes are illustrated in Figure 2. All geometries exhibit a surface pattern due to the slicing process, repeated laser paths and scaffold orientation in the VPP machine. SEM micrographs in Figure 2 also show a surface effect known as staircase, due to the approximation of manufactured scaffold with respect to the CAD design because of the fabrication of layers with a finite thickness.

### 3.2. Mechanical Tests

**Figure 3:**
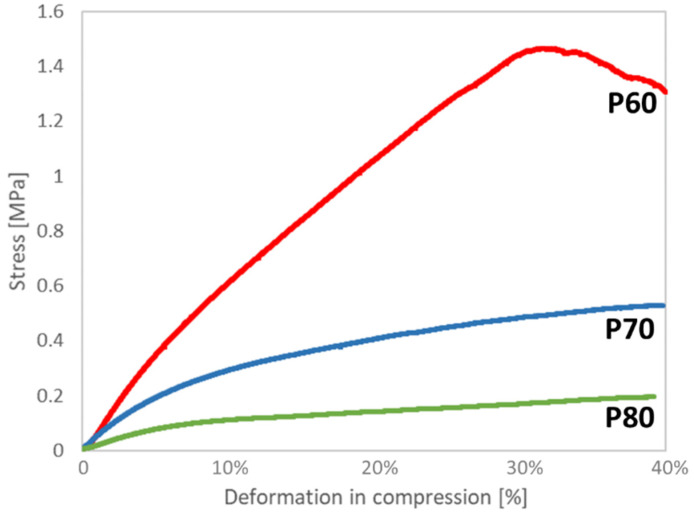
Experimental stress–strain curves of P60, P70 and P80 trabecular scaffolds.

The stress–strain curves from the compressive test are reported in Figure 3 for the P60, P70 and P80 trabecular scaffolds. The general trend of the stress–strain curves in Figure 3 shows a change in slope around 5% of the total deformation, where the mechanical behavior turns from an elastic to a plastic regime. This change can be ascribed to the progressive failure of an increasing number of struts reaching the yielding limit. This mechanism accounts for the dependence of the mechanical behavior of scaffolds on total porosity (Figure 3), which is determined by the number, length and geometrical arrangement of the struts in the trabecular structure. It is worth noting that the scaffolds recover their original geometry and size after mechanical tests.

Young modulus (E_S_) and ultimate compressive strength (σ_UCS_) values experimentally evaluated from the stress–strain curves are reported in Table 3 for the three different geometries investigated. Since the σ_UCS_ value of P60 perfectly matches with the middle and distal regions of the human mandible (Table 3), the mechanical behavior and biological performances were extensively investigated only for this geometry.

**Table 3:** Mechanical parameters of P60, P70 and P80 from compressive stress–strain curves. E_S_—Scaffold Young modulus; σ_UCS_—Scaffold ultimate compressive strength. Values of ultimate compressive strength of human mandibular trabecular bone in middle and distal regions by Misch et al. [22] are reported for comparison.

Scaffold Geometry	E_S_ [Mpa]	σ_UCS_ [MPa]
P60	8.5 ± 0.6	1.4 ± 0.1
P70	4.0 ± 0.5	0.50 ± 0.03
P80	1.6 ± 0.4	0.17 ± 0.03
Middle region including premolars of human mandible [22]	-	1 ÷ 4
Distal region including molars of human mandible [22]	-	0.5 ÷ 2.5

### 3.3. XµCT Analysis

**Figure 4:**
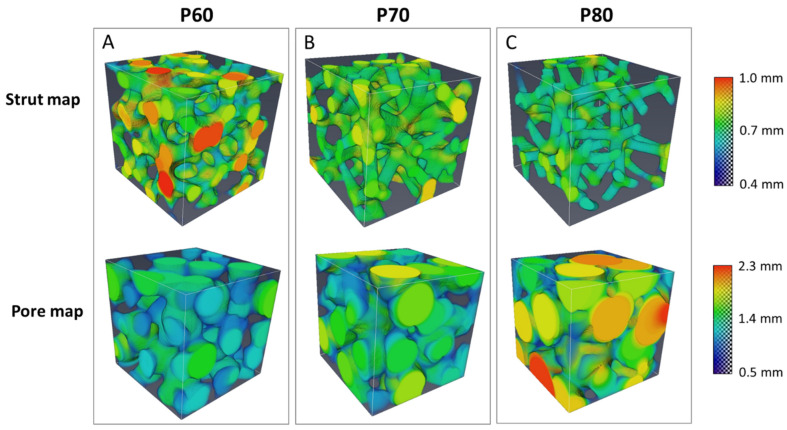
Strut and pore maps from XµCT analysis of P60 (**A**), P70 (**B**) and P80 (**C**) trabecular scaffold. Color scale-bars refer to strut thickness and pore size distribution.

Three-dimensional models of the trabecular scaffolds from XµCT analysis are displayed in Figure 4. The scaffold material distribution is plotted as a strut map, whereas the scaffold porous network is illustrated as a pore map, for the P60 (Figure 4A), P70 (Figure 4B) and P80 (Figure 4C) geometries. Furthermore, color scale-bars are reported in Figure 4 for strut thickness and pore size distribution. 

**Figure 5:**
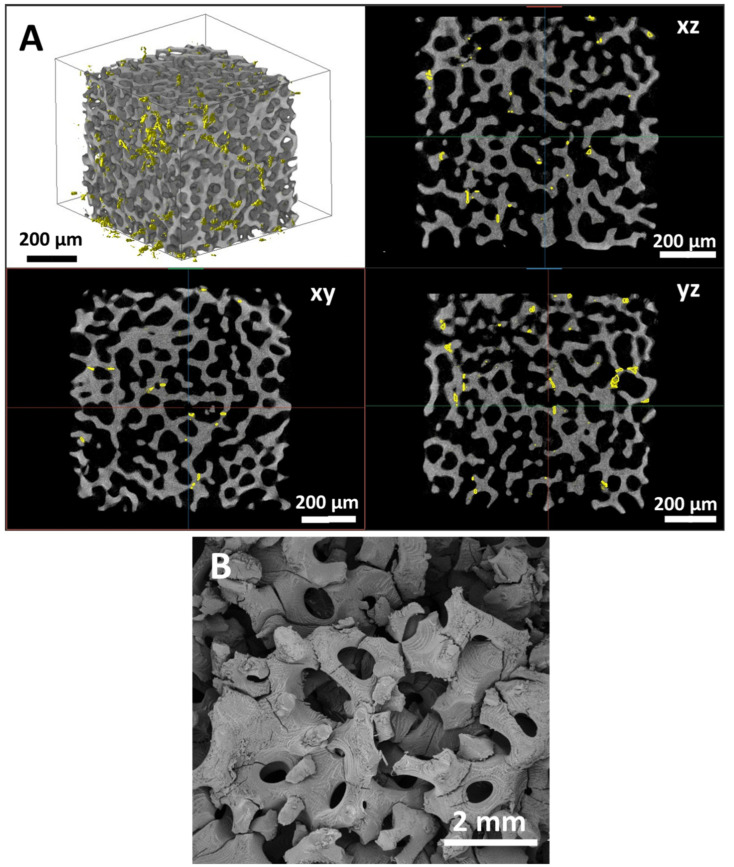
Strut failures in P60 scaffold geometry: (**A**) 3D reconstruction from XµCT and fractures visualization in xy, xz and yz planes—strut failures are highlighted in yellow; (**B**) SEM micrograph of scaffold surface after the compression test.

The specific surface, average strut thickness, average pore size and total porosity of scaffolds were quantified from the strut and pore maps before and after compressive tests (Table 4). From Table 4, it is evident that, within experimental errors, the global morphometry of scaffolds does not change after compression, despite the progressive failure of struts during the mechanical tests. Figure 5 shows the entity of strut failure after the compression test for the P60 geometry, as evidenced by XµCT analysis (Figure 5A) and by SEM observation of the scaffold top surface (Figure 5B). 

### 3.4. Biological Tests

**Figure 6:**
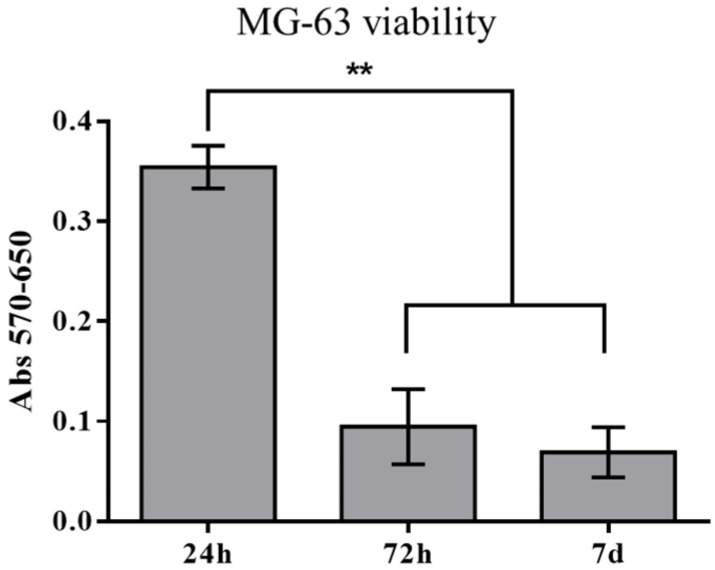
MG-63 viability at 24 h, 72 h and 7 days—data are expressed as absorbance (abs) at 570 nm with background subtraction at 650 nm—** *p* < 0.01.

The cell behavior on the P60 trabecular scaffold was evaluated at 24 h, 72 h and 7 days after MG-63 seeding. The MTT results suggest an intense reduction in MG-63 viability after 72 h of incubation, remaining unchanged up to 7 days of culture (Figure 6). 

**Figure 7:**
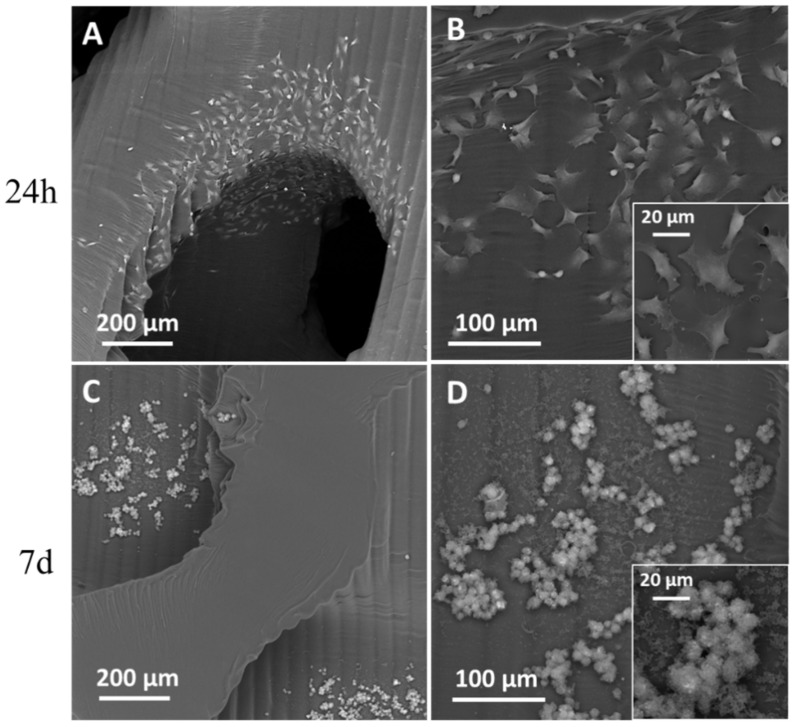
SEM observations of MG-63 on trabecular scaffold surface after 24 h from seeding (**A**,**B**) and inside scaffold after 7 days of culture (**C**,**D**). Insets in B and D show cells at higher magnification.

SEM observations (Figure 7) reveal that after 24 h from seeding, the cells were arranged preferentially close to pores (Figure 7A) and spread on the surface (Figure 7B), assembling several focal adhesions with the material (inset in Figure 7B). After 7 days of culture, a few groups of cells colonized the scaffold’s inner layer, migrating from the surface struts along the pore channels (Figure 7C). It is worth noting that SEM observations did not detect the presence of MG-63 cells on the scaffold surface after 7 days of cell culture. Moreover, MG-63 cells detected inside the trabecular structure showed apoptotic features (Figure 7D) with the loss of adhesion to the surface (inset in Figure 7D). 

### 3.5. PhC-XµCT Analysis at Synchrotron

**Figure 8:**
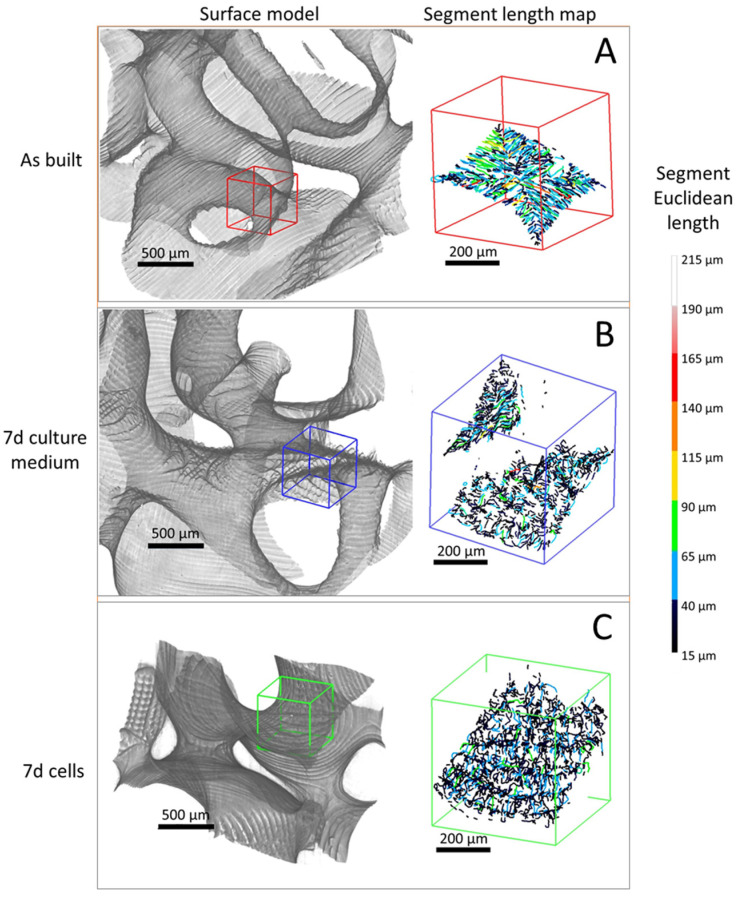
Three-dimensional surface model and corresponding segment length map of P60 trabecular scaffold as built (**A**), after 7 days in culture medium bath (**B**) and after 7 days of culturing with MG63 cells (**C**).

PhC-XµCT imaging was carried out on P60 scaffold geometry in three different conditions: (a) as built, (b) after 7 days in culture medium bath without cells and (c) after 7 days of culturing with MG63 cells. From the 3D reconstruction of the stack of 2D cross-sectional slices, MG63 cells fixed with osmium tetroxide became indistinguishable, likely because the enhancement of the scaffold surface signal due to the phase-contrast method hid the osmium tetroxide absorption contrast during PhC-XµCT acquisition. All scaffold typologies exhibit a surface micro-texture constituted of branches and nodes, due to the production process. The length of the segment connecting two branches or connecting a branch with an end node is the segment length of the surface micro-texture. Segment length maps of scaffold surface patterns are reported in Figure 8 for P60 in the three different conditions, (a) as built (Figure 8A), (b) after 7 days in culture medium bath without cells (Figure 8B,C) and after 7 days of culturing with MG63 cells (Figure 8C). From the segment length maps, the average segment length values are extracted (Table 5).

### 3.6. Correlation between Morphometry and Biomechanical Properties

**Figure 9:**
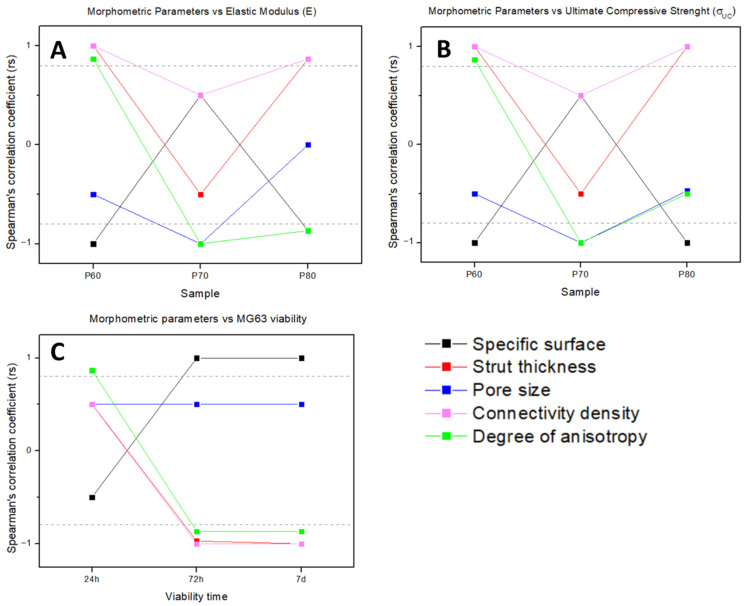
Morphometric parameters correlation with scaffold elastic modulus E_S_ (**A**), ultimate compressive strength σ_UCS_ (**B**) and MG63 viability (**C**), analyzed by Spearman’s correlation coefficient.

The Spearman’s correlation coefficients (SCC) between the features extracted from the scaffold analysis are summarized in Figure 9. Specifically, P60, P70 and P80 morphometric parameters from XµCT analysis were correlated with the elastic modulus (E_S_) and ultimate compressive strength (σ_UCS_), obtained from mechanical tests on the scaffolds. Moreover, the P60 morphometric parameters were correlated with the MG63 viability after 24 h, 72 h and 7 days of culture, according to the biological tests carried out on the P60 trabecular scaffold. The statistical inference based on SCC was focused on evidencing a general trend of the morphometric parameters mainly influencing the scaffold behavior, both in terms of mechanical (Figure 9A,B) and biological (Figure 9C) performances, according to the correlation coefficient (rs) value. |rs| > 0.8 (dashed line in Figure 9) is the optimal value for verifying a significative correlation between two parameters in the correlation matrix.

The mechanical values of the elastic modulus (E_S_) and the ultimate compressive strength (σ_UCS_) show a strong correlation with the same morphometric parameters. Specifically, in the P60 and P80 geometries, E_S_ and σ_UCS_ are strongly correlated with strut thickness, connectivity density (direct correlation) and specific surface (inverse correlation), whereas in P70, the most significative morphometric parameters for both elastic and plastic mechanical values are pore size and degree of anisotropy (inverse correlation).

Statistical analysis between scaffold morphometric parameters and cell viability (Figure 9C) considers the biological behavior of living cells found by an MTT assay at the different endpoints. From statistical analysis of biological data, after 24 h of cell culture on P60, only the degree of anisotropy demonstrates a strong direct correlation. Though, from 72 h up to 7 days of incubation on P60, it is worth noting that a general trend can be inferred suggesting the main morphometric parameters influencing MG63 viability are the same as P60 mechanical performances, with inverted proportionality. In particular, the specific surface (direct correlation), strut thickness, connectivity density and degree of anisotropy (inverse correlation) result from the main influencing parameters after 72 h from cell seeding. 

## 4. Discussion

In the scaffold-based approach for bone tissue regeneration, the control over morphometry allows for balancing scaffold biomechanical performances. In this experimental work, DS3000 trabecular scaffolds were produced by Vat photopolymerization (VPP) with the optimized printing parameters reported in Table 2. The trabecular geometry with target porosity and mean pore size was obtained through the generative design process illustrated in Figure 1, modulating only the number of seeds and strut thickness, unlike the three structural design parameters (strut diameter, unit distance and irregularity) adjusted by Du et al. [24] for developing irregular porous scaffolds for orthopedic reconstruction. Three different trabecular scaffolds were manufactured with 60% (P60), 70% (P70) and 80% (P80) total porosity, and increasing mean pore size, according to the values listed in Table 1. Trabecular geometries reconstructed in Figure 4 show thickness maps morphologically close to the bone microstructure obtained by Han et al. [7] for the trabecular part of the femoral epiphysis bone. The aim of the work is to identify the morphometric parameters which determine the mechanical and biological properties of trabecular structures in order to obtain useful indications to design a structure matching the requirements for tissue regeneration. Due to this, the inexpensive biocompatible material commercially known as DS3000 was found as a viable option for the production of trabecular scaffolds. 

From the SEM observations in Figure 2 and the 3D volume reconstruction from PhC-XµCT (Figure 8A), as-built scaffolds show a surface micro-texture formed of branches and nodes due to the slicing process, the repeated laser paths and the scaffold positioning in the VPP machine [25]. Conditioning for 7 days with culture medium affects the surface micro-texture (Figure 8B) by decreasing the average segment length (Table 5). However, as suggested by the segment length quantification results in Table 5, the cells’ adhesion and early viability (up to 7 days) on the P60 trabecular scaffold does not have an additional effect on the surface micro-texture morphology (Figure 8C), as also reported in Table 5. Thus, the culture medium acts as a smoothing factor for the surface micro-texture after already 7 days of conditioning, decreasing surface qualitative roughness, as also demonstrated by Amirikia et al. [26] after long-term exposure (up to 10 days) of silk fibroin in physiological medium. In our study, after only overnight conditioning, the surface micro-texture enables MG63 cell adhesion at 24 h from seeding (Figure 7A), healthy spreading on the scaffold surface (Figure 7B) by means of lamellipodia and filopodia (inset in Figure 7B). Furthermore, the porous network of the P60 trabecular scaffold allows cell distribution around pores and covering pore channels after 24 h of cell viability, as revealed by SEM micrographs in Figure 7A. The biological assessments on cell morphology and distribution after 24 h of incubation agree with Alvarez’s [25] findings on cell viability on DS3000 trabecular bone microarchitecture after longer-term incubation (from 72 h to 1 week), with cells adapting to the edges of the structure, displaying a spread shape on the structure and along the vertical walls of the pores. 

After 7 days of cell viability, the porous network of P60 allows the MG63 to penetrate the scaffold core (Figure 7C), despite the material causing a severe decline of cell viability after only 72 h of culturing (Figure 6), as also confirmed by the apoptotic morphology of cells detected by SEM (Figure 7D). A few groups of cells were able to migrate into the scaffold’s inner layers due to the proper design of trabecular geometry.

However, for longer-term viability, treatment of the scaffold surface is required to allow cell survival. Several authors in the literature improve the DS3000’s compatibility, reducing the harmful impact of byproducts, by coating the substrate with fibronectin, favoring the hydrophilic behavior of the material surface [19,25]. Other authors, such as Babi et al. [27] used dip coating with cellulose nanocrystals (CNCs) for tuning the nano-topography and functionality of 3D-printed scaffolds in DS3000. 

The scaffold’s mechanical behavior under compression, reported in Figure 3 as stress–strain curves, shows a plastic regime of up to 40% of deformation on initial scaffold height (Figure 3). However, the scaffold morphometric parameters such as specific surface, strut thickness, pore size and total porosity remain unchanged pre- and post-compressive test (Table 4), showing the elastic behavior of most struts during the compression test. Moreover, the failures of the struts reaching the yielding limit are clearly visible in the XµCT reconstructions and the SEM micrographs in Figure 5. 

When compressive load increases, struts parallel to the direction of the applied load are not subjected to shear stress, remaining in the elastic regime. On the contrary, inclined struts with respect to the applied load direction progressively reach failure because of the node displacement and shear stress component of the load. Thus, strut failures are multi-stage phenomena inducing a plastic regime. However, the number of struts reaching failure does not affect the overall elastic behavior of the trabecular scaffold, in contrast with the results of Baptista et al. [28] on polymer scaffolds (PLA) for trabecular bone replacement. The monotonic compression behavior of Baptista et al.’s [28] scaffolds is characterized by a linear elastic deformation of the porous structure, followed by a plastic collapse occurring when the local deformation within the struts leads to strut failure and then scaffold deformation. The scaffold reaches the compaction after strut failure saturation, leading to the gradual disappearance of the pores [28]. 

The experimental results of the mechanical tests clearly show that the P60 scaffold geometry perfectly matches the range of values of the ultimate compressive strength (σ_UCS_) of the middle region including the premolars and the distal region as well as the molars of the human mandible [22]. 

The performances of the three trabecular architectures are evaluated through the systematic quantification of morphometry as well as the mechanical and biological properties of scaffolds. The tuning of trabecular geometry by the generative algorithm in Figure 1 provides a wide design space to optimize the fitting to bone regeneration requirements in mechanical properties and permeability for cell and tissue in-growth. After Vafaeefar et al. [29], the most influencing morphometric parameters for scaffold biomechanical behavior are identified by statistical analysis (Figure 9). In P60, strut thickness, the degree of anisotropy and connectivity density are positively correlated with the modulus of elasticity, whereas the specific surface is inversely correlated (Figure 9A), according to the results of Vafaeefar et al., on a dual-lattice structure, similar to a Voronoi structure, which better captured the key morphometric parameters and mechanical properties of trabecular bone with respect to commonly used computational models (e.g., the gyroid and spinodoid structures) [29]. In addition, our experimental results indicate that the most influential geometric features for elastic modulus also show a strong correlation with the plastic deformation values (σUCS in Figure 9B), as well as with MG63 cell viability on P60 after 72 h from cell seeding, albeit with inverse proportionality (Figure 9C). Therefore, strut thickness, the degree of anisotropy, connectivity density and specific surface are the most significative morphometric parameters to balance for designing the biomechanical behavior of trabecular scaffolds for tissue engineering applications. 

In conclusion, the results of this experimental work clearly show that DS300 scaffolds generatively designed with trabecular geometry, produced by VPP technology with appropriate machining parameters, allow to meet the mechanical requirements of human mandibular trabecular bone and MG63 biocompatibility according to the standard specifications. In addition, from the correlation between the morphometry and biomechanical behavior of scaffolds, the most significative design parameters for bone tissue engineering applications are identified.

## 5. Conclusions

This experimental study aims to identify the morphometric parameters that mainly affect the mechanical and biological properties of trabecular structures, representing useful indications to design a device matching the properties needed for tissue regeneration. For such a reason, the low-cost biocompatible material commercially known as DS3000 was considered as a good candidate for the manufacturing of trabecular structures. The trabecular geometry was obtained through a generative design process, and the scaffolds were manufactured in DS3000 by Vat photopolymerization with 60% (P60), 70% (P70) and 80% (P80) total porosity. The performances of the three trabecular architectures were evaluated through the systematic quantification of morphometry as well as the mechanical and biological properties of the scaffolds. The main results obtained in this study can be summarized as follows: 

The mechanical behavior is governed by the elastic response of most struts, which allows the size and shape of scaffolds to be recovered after compression tests, despite the clear evidence of the failure of many struts;

The ultimate compressive strength values of P60 trabecular geometry match with the range of the middle region, including the premolars and the distal region as well as the molars of the human mandible, representing a useful implication for planning dental implant treatments and surgical placements;

Strut thickness, the degree of anisotropy, connectivity density and specific surface are the main influencing morphometric parameters to balance for designing the elastic and plastic behavior of trabecular scaffolds and their biological response, for tissue engineering applications.

## Figures and Tables

**Table 1:** Parameters used for the generative design of P60, P70 and P80 trabecular scaffold geometries.

Parameter	P60	P70	P80
Target porosity [%]	60	70	80
Target mean pore size [mm]	0.8	1	1.5
Number of seeds	2544	1397	759
Strut thickness [mm]		0.6	
Average strut length [mm]	0.6	0.8	1

**Table 2:** Process parameters used to produce DS3000 trabecular scaffolds by VPP.

Parameter	Value
Contours [n]	3
Hatching [mm]	0.07
Laser speed [mm/s]	5800
Laser power [mW]	86
Laser spot [mm]	0.04
Slicing [mm]	0.05

**Table 4:** Morphometric parameters of P60, P70 and P80 trabecular scaffolds pre- and post-mechanical compression test.

Morphometric Parameters		Before Mechanical Testing						After Mechanical Testing					
		P60		P70		P80		P60		P70		P80	
		AV	SD	AV	SD	AV	SD	AV	SD	AV	SD	AV	SD
Specific surface	mm^−1^	4.50	0.14	5.54	0.06	6.18	0.06	4.65	0.13	5.48	0.11	5.96	0.05
Strut thickness	mm	0.75	0.02	0.65	0.01	0.61	0.02	0.74	0.01	0.66	0.02	0.63	0.01
Pore size	mm	1.11	0.03	1.29	0.01	1.69	0.05	1.08	0.04	1.31	0.02	1.62	0.06
Total porosity	%	54	1	66	1	78	2	54	0	66	0	75	1

**Table 5:** Segment length values from segment length maps. AV—average values, SD—standard deviation.

Sample	As-Built		7 d Culture Medium		7 d Cells	
	AV	SD	AV	SD	AV	SD
Segment length (µm)	34.1	5.5	26.4	1.5	25.2	1.6

## Data Availability

Data sharing is not applicable to this article.
